# Calcium imaging and dynamic causal modelling reveal brain-wide changes in effective connectivity and synaptic dynamics during epileptic seizures

**DOI:** 10.1371/journal.pcbi.1006375

**Published:** 2018-08-23

**Authors:** Richard E. Rosch, Paul R. Hunter, Torsten Baldeweg, Karl J. Friston, Martin P. Meyer

**Affiliations:** 1 Wellcome Trust Centre for Neuroimaging, University College London, London, United Kingdom; 2 Developmental Neurosciences Programme, UCL Great Ormond Street Institute of Child Health, University College London, London, United Kingdom; 3 Department of Developmental Neurobiology & MRC Center for Neurodevelopmental Disorders, Institute of Psychiatry, Psychology and Neuroscience, King’s College London, London, United Kingdom; Oxford University, UNITED KINGDOM

## Abstract

Pathophysiological explanations of epilepsy typically focus on either the micro/mesoscale (e.g. excitation-inhibition imbalance), or on the macroscale (e.g. network architecture). Linking abnormalities across spatial scales remains difficult, partly because of technical limitations in measuring neuronal signatures concurrently at the scales involved. Here we use light sheet imaging of the larval zebrafish brain during acute epileptic seizure induced with pentylenetetrazole. Spectral changes of spontaneous neuronal activity during the seizure are then modelled using neural mass models, allowing Bayesian inference on changes in effective network connectivity and their underlying synaptic dynamics. This dynamic causal modelling of seizures in the zebrafish brain reveals concurrent changes in synaptic coupling at macro- and mesoscale. Fluctuations of both synaptic connection strength and their temporal dynamics are required to explain observed seizure patterns. These findings highlight distinct changes in local (intrinsic) and long-range (extrinsic) synaptic transmission dynamics as a possible seizure pathomechanism and illustrate how our Bayesian model inversion approach can be used to link existing neural mass models of seizure activity and novel experimental methods.

## Author summary

We show that Bayesian inversion techniques used in electrophysiological data are applicable to calcium imaging data derived from light sheet microscopy in the zebrafish brain. Using this approach we can now make inference on the underlying large-scale connectivity changes underlying pathological states such as seizures, and translate findings from zebrafish directly into the modelling frameworks utilised in human patients. Ultimately this modelling approach can be used to integrate evidence across different models of abnormal neuronal dynamics to facilitate a mesoscale understanding of seizure dynamics.

## Introduction

Epileptic seizures are transient disturbances in the brain’s electrical activity causing changes of patients’ behaviours or perceptions. Seizures have different causes, from gene mutations to acquired brain injuries [1]. The effects of particular pathologies on neuronal dynamics have been studied using animal models, where different interventions (e.g. chemoconvulsant exposure) can be evaluated *in vivo* [2–4]. Zebrafish in particular have been of recent interest for epilepsy research because they (i) are a vertebrate organism, (ii) allow the introduction of genetic mutations [5] and large-scale drug screening [6,7], and (iii) allow recording of neuronal function at high resolution across distributed brain networks [8,9]. There are now several studies of epileptic seizures in zebrafish [10–13] and recent imaging studies have captured network-wide changes in zebrafish brain activity during seizures [14,15]. However, a detailed mapping of how localised activity is integrated across the brain as a functional network during seizures is still missing.

Insights into seizure dynamics have largely been derived from computational modelling of EEG [16–18]. Using population models of neuronal activity allows the systematic description of the relationship between local brain circuit function and neuronal dynamics [19]. Combining novel empirical data and *in silico* models in this way has the potential to lead to an in-depth understanding of how specific disruptions at the microscale lead to whole brain phenotypes recognisable as epilepsy.

One strategy to combine computational modelling with imaging is dynamic causal modelling (DCM, [20]). Here, Bayesian model inversion is used to fit neuronal models to empirical data. This approach combines (i) widely-used neural mass models, and (ii) Bayesian model inversion algorithms. It is formally related to existing work on neural mass models in epilepsy [21–24]; as well as Bayesian inference approaches [25,26]. DCM has been widely applied to scalp EEG [27], invasive recordings in patients [28], and in invasive recordings from *in vivo* animal models [29].

Both EEG and LFP recordings are spatially sparse samples of distributed neuronal activity. Yet most modelling approaches assume measurable oscillations to represent homogeneous averages of population activity. Such averages can now be accessed more directly using light sheet microscopy, providing summaries of neuronal population activity that closely adhere to the modelling assumptions.

In this report we model empirical recordings of epileptic seizures in zebrafish across spatial and temporal scales using hierarchical DCM analysis: Spatial scales range from regional microcircuit neural mass models (mesoscale) to dynamic whole-brain networks (macroscale). Neuronal states of the underlying biophysical models capture fast oscillatory neuronal dynamics (millisecond temporal scale), whilst slowly varying model parameters capture the slow changes in the dynamic behaviour that occur over time (seconds to minutes temporal scale).

Seizures were induced with pentylenetetrazole (PTZ) in healthy larval zebrafish and recorded *in vivo* with light sheet microscopy of a single slice through the zebrafish brain capturing five main bilateral brain regions. PTZ is a well-characterised chemoconvulsant and acts as a GABA antagonist, thus disrupting inhibitory synaptic transmission. Acute seizures are believed to be associated with changes in (i) local microcircuit dynamics that allow for a (phase) transition between resting and seizure activity [19,30], and (ii) changes in whole-brain connectivity [31–33]. DCM allows concurrent testing of the following emerging hypotheses across these different spatial scales: (1) seizures lead to a measurable reorganisation of effective connectivity between regions [34], (2) local excitation-inhibition imbalance explains associated regional spectral changes [35], (3) in addition to changes in connection strengths, seizures are also associated with changes in synaptic transmission dynamics [29].

## Results

### Simulations

**Fig 1:**
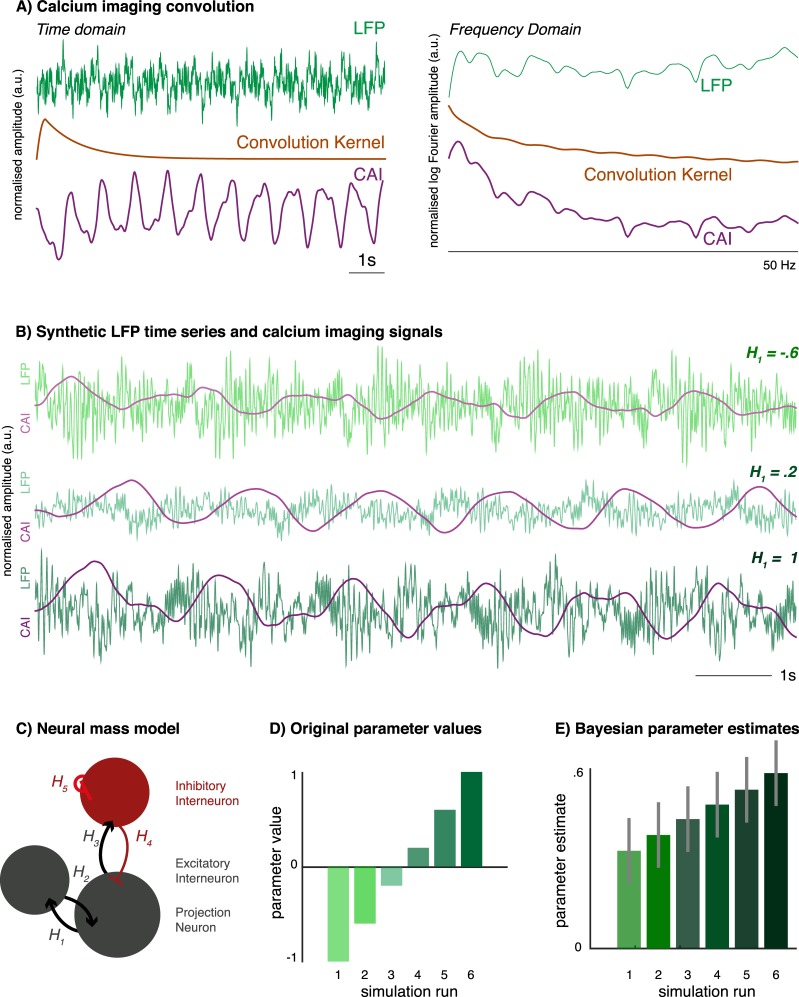
Dynamic causal modelling results of simulated calcium imaging time traces. (A) Left hand side time series show signal amplitude over time in arbitrary units. Calcium imaging dynamics were modelled by convolving LFP-traces (top) with a calcium imaging kernel (middle), resulting in a CAI time trace (bottom). The CAI trace follows slow LFP dynamics, whilst attenuating faster components of the original signal. Right hand side frequency plots show normalised log-amplitude derived from a Fourier transform of respective time series over a range of frequencies (1-50Hz). In frequency space, the convolution differentially scales low and high frequency components, but preserves most frequency features. (B) LFP-like time series plotted in arbitrary amplitude units over 10s. These are derived from a three-population neural mass model with increasing values of a single parameter, H_1_—also shown in *Fig 1C*. Example CAI traces after convolution are shown in darker colours. (C) A three-population neural mass model is used for generating LFP traces, and is subsequently fitted to the convolution-derived CAI traces. (D) Bayesian model comparison (Bayes factor 2.6) between repeated model inversions identifies correctly that differences between simulated CAI traces were caused by the effects of variations in the *H*_1_ parameter on the synthetic LFP traces. The parameter values included in the generative model are shown in the bar chart. (E) The DCM analysis provides estimates of the generative model parameters (shown in the bar chart). These results correctly infer the increase of *H*_1_ across the six model inversions from the CAI traces. Parameter estimates are shown here with a Bayesian 95% confidence interval (grey bars). Whilst the group mean parameter value and the effect size are different, this inversion correctly identifies the linear increase in the parameter from the simulated CAI dataset. *LFP*–local field potential, *CAI*–calcium imaging, *DCM*–dynamic causal model, *PEB*–parametric empirical Bayes.

In the analysis presented here, we used electromagnetic neural mass models originally designed to explain data features observed in LFP recordings. First, we confirmed the construct validity of this approach–i.e. applying DCM for local field potentials to time traces derived from light sheet imaging–by applying the analysis to synthetic data. These were derived from a neural mass undergoing predefined parameter changes: Using a single ‘source’ consisting of three coupled neuronal populations, we generate noisy LFP-like data. These are then convolved with a composite exponential decay kernel modelling calcium probe dynamics [36]. These surrogate fluorescence time traces are then downsampled to the sampling frequency achieved in the single-slice light sheet imaging (20Hz). This linear convolution equates to a simple addition of the signals in (log) frequency space. Because of the simple frequency composition of the calcium imaging kernel, this linear transformation preserves much of the spectral features in the underlying LFP like signal (**Fig 1A**).

The variations in the single neural mass model parameter introduces spectral changes in both the surrogate LFP and fluorescence time traces (**Fig 1B**). We fitted a three-population neural mass model (of the kind used to generate the LFP traces, **Fig 1C**) separately to each of the fluorescence time traces. This yielded six separate dynamic causal models (DCMs), one each fitted to the six timeseries generated using variations in a single parameter as shown in **Fig 1D**. Using a hierarchical parametric empirical Bayesian model, we then identified which parameter could best explain the differences in these DCMs (fitted to fluorescence signals). This successfully identified variations in the correct parameter (an intrinsic connectivity parameter *H*_*1*_) as the most likely cause for the differences in time series. Furthermore, the estimated between-DCM differences in *H*_*1*_ values also capture the direction of the linear change introduced in the original simulated LFP.

### Seizure recordings

**Fig 2:**
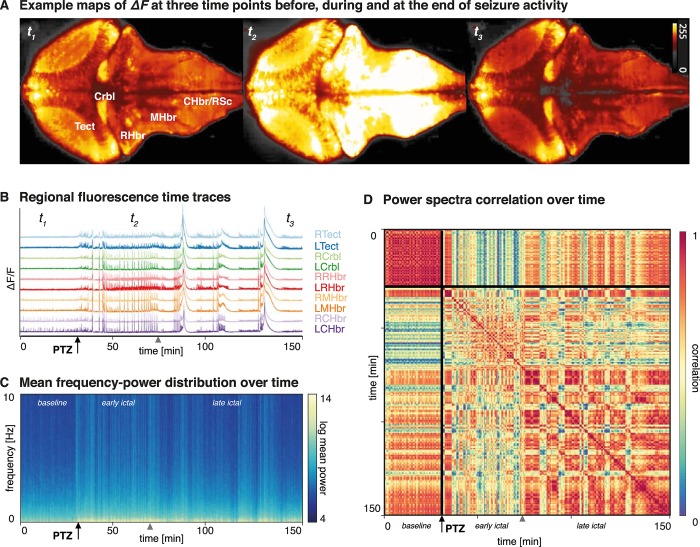
PTZ-iduced seizures recorded in the zebrafish larvae using light sheet imaging. (A) This image shows heat maps of fluorescence in a single slice of the intact larval zebrafish brain in the xy plane at different time points during the experiment (time points also indicated in *Fig 2B*). Seizure activity (t_2_) is visually apparent as an overall increase in neuronal activity compared to baseline state (t_1_). (B) Regionally averaged time traces of the fluorescence signal across 5 bilateral anatomically defined regions are shown for the whole duration of the experiment in a single animal (150 minutes). Seizures are readily apparent as an inrease in generalised and apparently synchronous high amplitude activity. (C) Average Fourier power spectra across fish and across all brain regions are plotted against time for the duration of the experiment, using a sliding window estimator (length: 60s, step: 10s), with colours indicating log-power. The graph is a the average over *n = 3* fish. PTZ causes an increase largely in low frequencies (<2Hz), with intermittent bursts of more broadband activity. (D) A correlation matrix showing correlation indices of the power-distribution patterns across different time points (delay-delay matrix). This reveals three distinct time periods, corresponding to baseline (<30min), ictal (30-70min) and late ictal (>70min) phases with distinct spectral signatures and temporal dynamics. Tect—Tectum, Crbl—Cerebellum, RHbr—Rostral Hindbrain, MHbr—Mid-Hindbrain, CHbr/RSc—Caudal Hindbrain/Rostral Spinal Cord.

In order to elicit epileptic seizures, PTZ was infused in the bath of *n = 3* zebrafish larvae. The resultant seizure activity was recorded with light sheet imaging utilising a genetically encoded calcium sensor (GCaMP6F). Neural activity was recorded *in vivo* in agarose immobilised larvae capturing a single slice of the intact brain. The changes in activity within the whole imaged slice was readily apparent in the fluorescence images (**Fig 2A**). We divided the slice into 5 bilateral regions of interest to extract fluorescence time series from the recording. These showed distinctive features consistent with highly correlated epileptic seizure activity (**Fig 2B**). Using a sliding window (length: *60s*, step: *10s*) we could estimate the time-changing frequency content using a Fourier transform, which demonstrate a particular increase in low frequency power after PTZ infusion (**Fig 2C**), with additional intermittent bursts of broadband activity seen. Estimating correlations between the regional power-frequency distributions across different time windows reveals apparently distinct phases of PTZ induced seizures (**Fig 2D**): A baseline that is stable over time (0–30 minutes), an initial ictal period that differs most from the baseline state (30–70 minutes), and a late ictal period where time periods of apparent similarity (i.e. high correlation) to the baseline are interrupted by intermittent different (i.e. low correlation) segments (70–150 minutes).

### Functional network architecture at baseline

**Fig 3:**
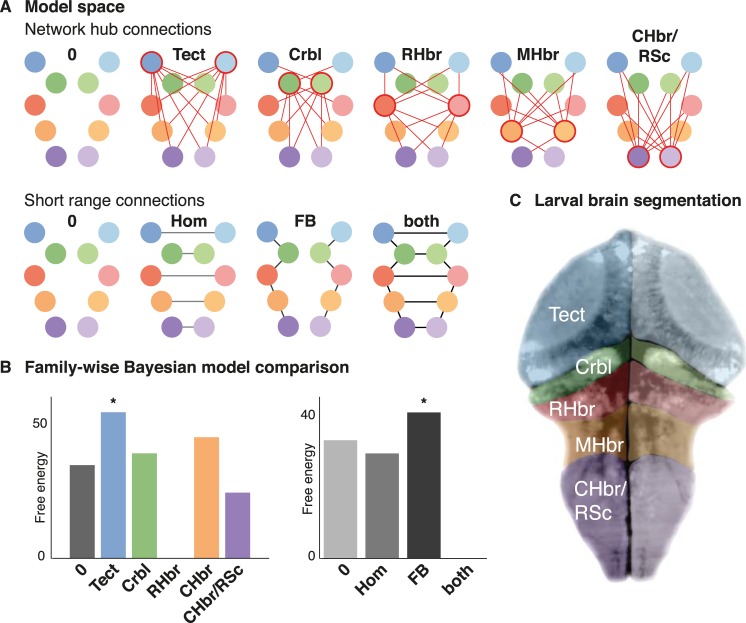
Network model architecture during interictal background activity. (A) Two aspects of a factorial model space are shown: extrinsic connectivity of putative network hubs (yielding 6 types of models), and short range connection between neighbouring and homotopic nodes (yielding 4 different types of models); a total of 6 * 4 = 24 models were evaluated, where any one model is combines one of the network hub connectivity architectures with a short-range connectivity setup. Bayesian model reduction was used to estimate the model evidence across this model space characterised by the presence or absence of these defined sets of between-region reciprocal connections (neighbouring, homotopic, and hub connections). (B) For each model family (corresponding to the factorial model space), the free energy difference to the worst-performing model is shown. In DCM, the free energy difference is used to approximate model log-likelihood differences: Asterisks indicate the winning model family identified from Bayesian model selection. These results indicate that the model with neighbouring, and homotopic connections as well as the optic tectum with hub-like connectivity best explain the observed spontaneous activity at baseline. (C) Mapping of the ROIs for this analysis is illustrated as overlay on a single fluorescence image taken from one of the animals included in this study. Areas were identified based on visible neuroanatomical landmarks and correspond to the nodes of the same colour in the network representations of the model space.

We employed Bayesian model comparison to identify the effective connectivity network that best explains the baseline data. In brief, baseline activity was modelled as spontaneous activity arising from a coupled network of neuronal sources. Each source is made up of a three-population neuronal microcircuit (excitatory and inhibitory interneuron populations, as well as a main projection neuronal population) that is fitted to a cross-spectral density summary of the fluorescence signal at baseline. A single fully connected network was fitted to an average of the baseline activity by inverting a single fully connected dynamic causal model (DCM). Using Bayesian model reduction and Bayesian model selection we compared models, where specific sets of between-region reciprocal effective connections were either present or absent. These sets of connections were (1) hub-like connectivity between any one region and all other regions; and (2) short range connection between neighbouring, and homotopic brain regions (**Fig 3A**). Bayesian model comparison across the reduced models in this model space provided evidence that the baseline configuration can best be described as a network of neighbouring connected nodes with the tectum acting as a network-wide hub (**Fig 3B**). Notably in this mesoscale modelling, such directed connectivity is understood to be the average influence one region has over another–this may be mediated monosynaptically or through additional (hidden) network nodes. In the model each source contains a simply parameterised steady state noise input function that is updated as part of the model inversion–therefore synchronous oscillations between different nodes could possibly be explained away during the inversion by fitting identical input functions to each source. Where more complex models with specific connectivity patterns are identified as the most parsimonious explanations for the particular spontaneous activity, this suggests that not all aspects of the complex cross-spectral densities (which include phase differences between sources) can be explained by common input alone.

### Hierarchical dynamic model of seizure activity

**Fig 4:**
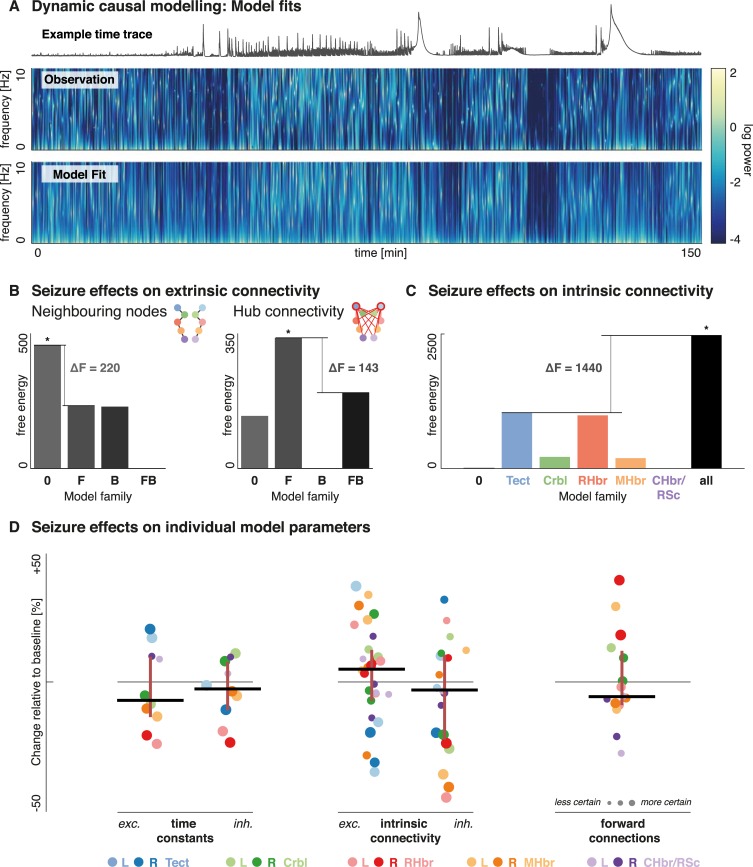
Group level effects of PTZ-induced seizures on synaptic coupling. (A) Here (1) an example fluorescence time trace from a single region, (2) an example eigenmode summary of the cross-spectral density changes over time observed across all region in a single fish (this is derived from a multivariate autoregressive model and constitutes the primary data features in DCM), and (3) the model fits of windowed DCMs to that same animal are shown. The middle and bottom panel both plot frequency power distribution across the time of the experiment, where the log-power for any given frequency is represented by colours corresponding to the same colourbar (range -4 to 2). DCMs fitted to these individual time windows capture the spectral changes measured well for the duration of the experiment. (B) Free energy approximation for the model-family evidence for reduced models where PTZ-induced changes were restricted to a subset of coupling parameters is shown. Bayesian model comparison at this second (between time-window) level was performed to compare reduced models with PTZ-induced changes in *F* forward, *B* backward, *FB* both, *0* or neither type of regional connectivity. Asterisks indicate the winning models. Only changes in connections from other brain regions to the hub region show evidence of being modulated by the seizure activity. (C) Similarly, free energies for model families that allow for intrinsic connectivity parameter changes in none of the brain regions, single brain regions, or all brain regions are shown. The asterisk indicates the winning model. There was strong evidence for intrinsic connection changes in all brain regions. (D) Estimates of the PTZ-effect on DCM model parameters are shown, corresponding to the expected change relative to baseline that was induced by PTZ. Each dot represents a posterior density, centred around the expected value, and its size inversely correlated to the covariance (or uncertainty), i.e. the larger the dot, the more precise the estimate. Dots are colour-coded by region as shown in the legend. Lines indicate the median of the expected values with whiskers showing 25^th^ and 75^th^ centiles respectively–but note that individual parameter estimates are not random samples from an underlying distribution but themselves represent more or less precise model parameters fitted to the observed data. ***Model families (extrinsic)*:** 0 –no extrinsic connectivity changes; F–extrinsic connectivity changes in forward connections only; B–extrinsic connectivity changes in backward connections only; FB–extrinsic connectivity changes in both forward and backward connections. ***Model families (intrinsic)*:** 0 –no intrinsic connectivity changes; Tect–intrinsic connectivity changes only in the bilateral optic tectum; Crbl–intrinsic connectivity changes only in the bilateral cerebellum; RHbr–intrinsic connectivity changes only in the rostral hindbrain; MHbr–intrinsic connectivity changes only in the mid-hindbrain; CHbr/RSc–intrinsic connectivity changes only in the caudal hindbrain/rostral spinal cord; all–intrinsic connectivity changes across all areas.

Using this model architecture, individual DCMs are fitted to the sequence of sliding-window derived cross-spectral density summaries of the original data. Spectral changes were found to be consistent across the fish used for this study (**S2 Fig**). All seizure effects are subsequently assumed to arise from variations in the model parameters that were estimated from the baseline architecture. Thus the seizure activity may ‘switch off’ connections (through reduction of the particular parameter), or silence a node in the network (through increases in self-inhibition), but no new connections or nodes are added to explain data features that arise during the seizure. At this stage (i.e. first level models), each time window is modelled as an independent DCM. The model fits show that these independently inverted models recreate the dynamic fluctuations of spectral composition observed during a seizure very well and thus provide a good representation of the original data features (**Fig 4A**). Across all complex cross spectra (for all time window and all animals), the model fits explain 74.6% of the variance in the original data (R^2^ = 0.746).

**Fig 5:**
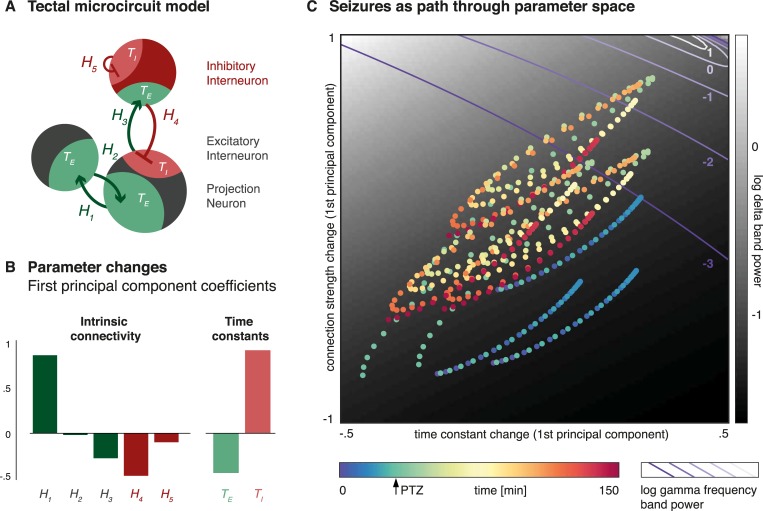
Temporal evolution of intrinsic coupling parameter changes in the optic tectum throughout the seizure. (A) A single source 3-population model is shown, indicating the seven parameters that are fitted as part of the dynamic causal modelling: 5 intrinsic connectivity parameters (*H*_*1*_ *–H*_*3*_ excitatory connections, to *H*_*4*_ *–H*_*5*_ inhibitory connections), and 2 time constants (*T*_*I*_ and *T*_*E*_). (B) A principal component analysis was performed separately across the posterior estimates of intrinsic connectivity, and time-constant parameters for the optic tectum across all time windows of the experiment. The coefficients for the first principal component of intrinsic connections (left) and time constants (right) are shown here. (C) Using these two principal components, parameter estimates of intrinsic coupling within the optic tectum for each individual time window are projected onto a two-dimensional parameter space. Each point of this projection is colour coded according to its time in the experiment from which the estimate was derived. In order to relate location in parameter space to spectral output at the optic tectum, for each point in this parameter space, we ran a dynamic causal model of the optic tectum in simulation mode to yield an yield an estimate of power spectral densities at that particular parameter combination. Here we map the predicted mean log-power in the delta- (black and white heat map) and gamma-band (purple isoclines) respectively. Thus the figure shows the temporal evolution of intrinsic coupling parameter estimates within the optic tectum during the seizures on a map of the spectral energy for different frequency bands for the specific parameter combinations. Time points just after PTZ injection occupy the most extreme top-right corner of this parameter space. This indicates both slower inhibitory connectivity (time constant component) and stronger excitatory / weaker inhibitory connectivity (connectivity time constant component). These paramete changes are associated with high powers in both the gamma and the delta band.

Parametric empirical Bayes (PEB) can be employed to identify parameters across individual DCMs that vary systematically with specified experimental variables. In brief, PEB allows one to invert hierarchical models where, in this instance, the first level of the model corresponds to a sequence of time windows. The second level of the model then uses the posterior densities over the first level parameters to model changes (here fluctuations) in the first level parameters. We modelled PTZ induced changes as a mixture of four effects: (1) a simple model of PTZ bioavailability as first order pharmacokinetics with a maximum effect achieved at 30 minutes, (2) a tonic effect switched on for the duration of PTZ exposure, (3) a monotonically increasing effect representing the influence of prolonged seizure activity, (4) oscillatory effects at different slow frequencies represented by a set of discrete cosine transforms [37]. This approach provides a single model at the group level (i.e. across all time windows, and all individual fish) and parameter changes are modelled as a mixture of experimental and random effects. The estimated mixture of parameter effects yielded consistent spectral changes across the individual fish used for the study (**S3 Fig**). This type of modelling assumes that discrete oscillatory neuronal states (e.g. apparently distinct states during the seizure with very different neuronal signatures) arise from mostly smooth fluctuations in the underlying parameters (smooth transitions are tracked in **Fig 5**; as well as **S4 Fig**). This indeed is a feature of the types of models at the heart of the dynamic causal modelling (i.e. neural mass models) and their nonlinear mapping between parameters and states. This has been exploited extensively in the past to link apparently sudden transitions in neuronal dynamics to slow synaptic or neurochemical changes [19] that could cause them. These second level inversions also provide an estimate of the model evidence, so that different models can be tested against each other.

In the first instance, we compared models where only subsets of between region connections were allowed to vary between time points. Bayesian model comparison shows that only changes in the forward connections to the network hub (i.e. bilateral tectum) are required to explain the spectral changes during seizure activity (**Fig 4B**). Model comparison was also used to test for PTZ induced changes in the intrinsic coupling parameters in individual regions. There was strong evidence for an involvement of all measured brain regions (**Fig 4C**). Note that among the models where seizure activity only affects intrinsic connections of a single node, the tectum and rostral hind brain emerge as the most likely models–suggesting that variations in both have a particular impact on the seizure dynamics. The estimated parameter changes induced by PTZ were varied between different brain regions, but overall showed a relative reduction in excitatory time constants (suggesting faster responses), reduction in inhibitory intrinsic connections, and a reduction the influence of other regions on the optic tectum (i.e. a reduction in forward connections; estimates of the time varying parameters shown here summarise effects at the assumed peak PTZ effect time window early in the seizure **Fig 4D**). Most of the largest effects in terms of intrinsic model parameters affect the rostral hind brain and the optic tectum, with at times apparently opposing effects (e.g. opposing changes in excitatory time constant changes). Future studies may explore the differences in dynamic responses to PTZ stimulation across these regions.

Note that each value on this plot represents a posterior density that consists of both the estimated parameter value for the particular parameter, and a posterior covariance that represents the uncertainty around that parameter estimate. In dynamic causal modelling, inferences are made via model comparison (i.e., log evidence or odds ratios provided in **Fig 4**). Thus **Fig 4D** provides a quantitative characterisation of the underlying effect sizes in terms of posterior densities, under the best model. The values in Fig 4 shows the effects and between region differences (the scatter of the dots reflects precise and systemic inter-regional differences, not random effects). Whilst similar parameters are grouped in the scatter plot for visualisation purposes, they represent different aspects of the same model inversion and thus the optimal model fit, given the data. As such their mean or median value is only informative to provide an intuition as to the overall direction of the effect. The intuition of how individual parameter changes relate to spectral output is characterised in more detail for one region (the optic tectum) below (**Fig 5**).

In a next step, we quantified the temporal evolution of the parameter changes in one example region so as to map (smooth) parameter changes to associated changes in the spectra over time. For this we collated all the parameters intrinsic to that region (i.e. intrinsic coupling parameters and time constants) and simulated the associated spectral output from a single three-population source. This was done for a range of different parameter values informed by the empirically-derived posterior parameter estimates from the PEB analysis above (**Fig 5A**). We then extracted the parameter estimates for time constants and intrinsic connectivity within the right tectum over time across all components of the PEB model (i.e. tonic seizure effects, monophasic PTZ effect, prolonged seizure effect, discrete cosine transforms, random between-animal effects). In order to visualise the parameter changes over time, we derived a low-dimensional representation of the data: We extracted the first principal component of the posterior estimates of the intrinsic connectivity parameters, and the time constants over time (**Fig 5B**). The first principal component of the time constant changes explains 70.9% of their variance; the first principal component of the intrinsic connection changes explains 49.2% of their variance.

Plotting each time window onto this reduced parameter space containing most of the variance in the coupling parameters represents the seizures as a spiral path through parameter space. We can apply the parameter combinations at each point in the parameter space to a microcircuit model and predict the spectral output. Here we show log delta band power as a heat map, with log gamma band power superimposed as isoclines (**Fig 5C**). This forward modelling approach shows that during the seizure, the model enters a section of parameter space characterised by both high delta and gamma power components, which is also seen in LFP recordings during seizures in zebrafish reported in previous studies [6]. In **S4 Fig**, we added an additional component for the intrinsic connection as a third dimension, with the two combined now explaining 80.0% of the intrinsic connectivity variance, and revealing a separation of the distinct seizure phases identified from the spectral analysis alone (**Fig 2**).

## Discussion

Even well studied pharmacological interventions, such as PTZ show multi-scale effects across the nervous system [33,38–40]. Thus, linking membrane-level changes with the whole-brain seizure phenotype remains challenging. Here, we combine zebrafish light-sheet imaging during PTZ-induced seizures with dynamic causal modelling in order to identify network-wide connectivity changes.

### Validity of DCM for calcium imaging traces of seizure activity

Calcium imaging time series are highly correlated with concurrent LFP recordings [41]. Whilst LFP generally allows measuring of neuronal population activity at a higher temporal resolution (including activity >100Hz), calcium imaging is more limited due to both the sampling frequency [42], and the fluorescence decay dynamics of the calcium-sensitive probe [36].

The predominant frequency components of both resting brain activity and seizure activity in the larval zebrafish brain are in the delta (<4Hz) and theta (4-8Hz) band [10]. Neuronal fluctuations in these frequency bands are largely preserved in calcium imaging, and apparent even at sampling frequencies as low as 20Hz. Our simulations illustrate the construct validity of using neural mass models that generate electrophysiological responses to explain calcium imaging data: DCM allows correct causal inference from calcium fluorescence time series to underlying coupling parameters. This approach provides deeper neurobiological insights than functional connectivity approaches alone. Furthermore, our hierarchical modelling allows tracking of slowly varying model parameters [29], offering explanations for qualitatively very sudden changes in oscillatory behaviour (represented by the output of individual DCM models) emerging from gradual changes in model parameters (represented across-DCM parameter changes estimated in the PEB approach).

The DCM analysis of simulated data here only recovered the trend of the activity (not the actual value). We use a convolution kernel to simulate the effects of calcium imaging on time series, but we inverted the models with a DCM without a kernel, hence some difference is anticipated. However, in the time-resolved analysis of connectivity changes during a seizure, we are interested in the relative change of model parameters over time more than the background setup (which we account for as an additional group-mean effect in the hierarchical modelling with PEB-DCM).

This analysis harnesses specific advantages of regionally averaged calcium imaging: Light-sheet microscopy samples in a spatially unbiased fashion, thus providing a closer approximation to the assumptions underlying neural mass models [43]. Heuristically, this spatial averaging suppresses local fluctuations in the same way that averaging over time in event related potential studies (in electrophysiology) reveals dynamics that are conserved over multiple realisations. Furthermore epileptic seizures are an emergent property at the level of neuronal populations, and computational models specifically addressing this ‘mesoscale’ may yield important insights about emergent population-wide features less readily apparent from microscale modelling of individual neurons [44]. Furthermore, our analysis allows inferences to be linked back to established knowledge about the anatomical regions included in the study. However, we do not fully exploit the spatial resolution offered by the calcium imaging data, which will need to be addressed in the future with scalable custom approaches to modelling of individual neurons [45,46]. One strategy to exploit the resolution of light sheet images is through definition of ‘regions’ based on microscale neuronal properties (e.g. correlated activity, distribution of neurotransmitter receptors [47])–whilst the same model inversion technology illustrated here remains applicable, the data features selected for DCM would be informed by the neuroanatomical and neurophysiological information in the light-sheet imaging data and thus exploit the spatial resolution.

### Network organisation in the larval zebrafish brain

DCM allows for the estimation of network coupling parameters underlying neurophysiological recordings, within the constraints of the available data and the hypothesised model space. The first step of our analysis thus aims to explain the pre-ictal baseline fluctuations in 5 bilateral brain regions of the zebrafish brain through any one of the proposed model architectures. Both changes in the data used for further analysis (e.g. changes in the regional divisions, or extension beyond a single imaging plane) and changes in model space (e.g. inclusion of another possible hypothesis) may therefore impact on the inference drawn. However, both the data included in this study and the model space explored reflect the types of hypotheses we sought to explore.

Early during zebrafish development, retino-tectal connections develop, and stereotyped but effective visuomotor behaviour is established [48–51]. This is associated with distributed network activity involving information flow from the optic tectum to other brain areas. This visually-dominated early network activity is also apparent in the DCM analysis, where the tectum has been identified as a hub with widespread connectivity to the rest of the larval zebrafish brain from resting state light sheet recordings at baseline.

This network organisation is modulated during seizure activity, where our modelling identifies a reduction of the effective forward connections from other brain areas to the optic tectum. This asymmetric shift in connectivity (with only forward, but not backward connections affected), may be indicative of a key role of the optic tectum—as a central network hub at baseline—in driving network-wide synchronisations during an epileptic seizure. The selective reduction in effective connectivity corresponds to previously reported seizure-related changes in functional connectivity estimated from human EEG recordings, where increased clustering during a seizure has been described [52].

Fluctuations in effective connectivity between regions is usually thought of as resulting from changes in direct synaptic connectivity [53]. Where all connections towards a single brain region are involved, this may be due to (i) specific synaptic mechanisms affecting synaptic receptors at this particular region, or (ii) changes in local excitability. However, the asymmetric involvement of a single brain region–where only effective connectivity *to* (and not *from*) the optic tectum is reduced–suggests that local microcircuitry changes may underlie the macroscale changes. The relationship between local and macroscale network changes in epilepsy in the context of hierarchically coupled brain areas is discussed elsewhere [31]. This phenomenon has been formally described in other modelling work through a slow local permittivity variable that governs synchronisation between different brain regions and represents different slowly unfolding changes in local energy and metabolic milieu [54].

### Intrinsic coupling changes disrupt excitation-inhibition balance in the temporal domain

PTZ acts as an acute chemoconvulsant in a range of different model organisms, likely due to allosteric inhibition of GABA-A receptors [38]. Previous work on a PTZ rat model showed dose-dependent regionally specific cellular activation [33], suggesting differential susceptibility of different brain regions to PTZ effects. Bayesian model comparison of seizures recorded from the zebrafish in this report indicate that PTZ-induced changes of intrinsic neuronal population coupling were required in each of the brain regions. From the free energy distribution across models with different single regions affected by seizure changes, we found relatively high model evidence for models comprising seizure-related parameter changes in the optic tectum, or in the rostral hindbrain, suggesting that there is heterogeneity in the contribution of individual brain regions to the evidence for the winning model.

PTZ-related seizure effects here are modelled under the assumption that they arise from changes in the existing extrinsic (between-region) connections and intrinsic coupling parameters. We expected most of the interesting effects to occur on the coupling parameters within regions (as most of the PTZ effect will affect local inhibitory interneuron connectivity [55]). Whilst epileptogenesis in the brain (i.e. developing the propensity for recurrent seizures) may require the establishment of novel, pathological connectivity, acute seizure activity most likely will not. Thus, our modelling approach has the ability to account for most neurobiologically plausible mechanisms underlying acute seizures.

However, these effects varied widely between regions. This in part reflects different baseline configurations of the regional source models, which in turn require different shifts in coupling parameters. Yet, overall the PTZ-related changes are broadly consistent with our current understanding of its effects at the neuronal membrane. Specifically, PTZ is expected to cause a relative decrease of inhibitory connectivity compared to excitatory connectivity; and preferential blockade of fast GABA-A (and not GABA-B) mediated transmission would be expected to cause an increase in the relative inhibitory transmission time constants (i.e. slowing down), compared to excitatory synaptic dynamics–both of these effects are observed in the parameters estimated across the whole brain slice here (noting that population-level time constants are likely a product of several convergent synaptic effects [38,56,57]. Left-right asymmetries in the intrinsic estimated connectivity in the optic tectum is most likely secondary to differences in light stimulation received by either eye.

Further exploration of individual parameter effects at a single brain region supports the notion that seizure dynamics in this recording are largely caused by two main effects: a relative disturbance in excitation / inhibition balance with increased excitation and decreased inhibition, and a reciprocal disturbance in the dynamics of excitatory and inhibitory connectivity with slower inhibition and faster excitation. Because we have fitted fully generative neural mass models, we can make predictions about the spectral output caused by particular parameter combinations beyond the measured ≤10Hz frequency range. This approach reveals that particularly the time points where both connectivity and time constant effects changes reach their respective extremes, the typical seizure spectral output containing high amplitudes in both low (i.e. delta) and high (i.e. gamma) frequency components emerges. The addition of PTZ causes an increase in broadband activity, with particularly high predicted power in the gamma band early after PTZ administration, and more pronounced increases in slow frequency power as the seizures evolve. This is consistent with previous studies that have separately recorded LFP traces during seizures in zebrafish [6,14].

Recurrent neuronal loops with a close balance of overall excitation and inhibition underlie spontaneous brain activity. The brain is believed to operate near a transitional state from which both subcritical, random dynamics and supercritical, ordered dynamics can emerge (i.e. self-organised criticality, cf. [58]). Blocking of the largely GABA-A mediated local recurrent inhibition shifts this balance and allows ordered, seizure-like activity to occur [59]. In our model the emergence of seizure dynamics requires changes in both connection strengths and their temporal dynamics. Future research will address how different pathologies may converge on the mechanisms that underlie observable seizure dynamics.

### Conclusion

The analysis presented here illustrates the use of computational modelling to explain neuronal dynamics in the larval zebrafish brain during acutely induced seizures. This approach exploits the spatial independence of single plane *in vivo* light-sheet recordings of brain regions and uses dynamic causal modelling to identify the mechanisms underlying seizure dynamics. Our Bayesian model inversion scheme allows translating observations from whole-network novel light sheet imaging to the concepts and models used to explain electrophysiological abnormalities observed during seizures.

Seizures in this model are associated with an asymmetric decoupling of the network hub, and changes in excitation/inhibition balance that crucially also involve the temporal dynamics of excitatory and inhibitory synaptic transmission. Mapping the expected spectral changes along both the connection strength and time constant domains of the model within the pathophysiological range estimated from acute seizures allows us to delineate the independent contribution of changes in either type of parameter to the overall dynamics. This is the first step to establishing network-wide mechanisms that underlie seizures and may be targeted with novel treatments for epilepsy.

### Limitations

Like all Bayesian modelling approaches, DCM only provides estimates of the likelihood of individual models in direct comparison to a larger model space. As the model space evolves, and other plausible hypotheses are being tested, a new model may offer an overall better solution to the inverse problem. Furthermore, as our understanding about the underlying neurophysiology progresses, prior knowledge can be incorporated into the model inversion (quantitatively in terms of changes in the prior parameter expectations) and thereby nuance Bayesian model comparison.

It is also worth noting, that the DCM results are ‘true’ in that they represent the mathematically simplest approximation of a given dataset under specific assumption–a more complex model may be biologically implemented but not emerge as the winning model because the added complexity is not required to explain the particular data features at hand.

The approach presented here illustrates how light-sheet imaging in zebrafish larvae can offer an insight into the kind of mesoscale dynamics that are also observable (and of interest to the modelling communities) in electrophysiological recordings. The type of modelling and inversion scheme used here is flexible enough to ultimately accommodate data that contain some of the microscale information about the neuronal ensemble (e.g. by defining ‘regions’ through molecular markers present on individual neurons rather than gross anatomy), however this was beyond the scope of the current–proof of concept–paper. Furthermore, our imaging protocol was optimised to capture widespread activity changes at high sampling frequencies (e.g. by only imaging a single plane), assuming that activity in this plane reflects the dynamics of the whole region, whilst excluding non-imaged regions (that are situated above or below to the plane) from the analysis.

Whilst only a small number of fish were included in this analysis, the effects at the level of the recordings are large and consistent between fish. For future studies on more subtle effects and observations (e.g. the topological organisation of spontaneous seizures), a higher number of fish is likely to be required.

## Materials and methods

### Ethics statement

This work was approved by the local Animal Care and Use Committee (King’s College London) and was performed in accordance with the Animals (Scientific Procedures) Act, 1986, under license from the United Kingdom Home Office.

### Experimental model and subject details

#### Zebrafish Maintenance

Zebrafish were maintained at 28.5°C on a 14 h ON/10 h OFF light cycle. Transgenic line used: Tg(elavl3b:GCaMP6F) [60]. Sex of individual animals not known.

### Method details

#### Construction of Light-sheet microscope

The light-sheet design was based on that described in [61]. Briefly, excitation was provided by a 488nm laser (488 OBIS, Coherent) which was scanned over 800μm in the Y direction of the illumination plane by a galvanometer mirror (6215H/8315K, Cambridge Technology) creating an illumination sheet in the XY-plane. The sheet was associated with two pairs of scan and tube lenses, scanned along the z-axis using a second galvanometer mirror (6215H/8315K, Cambridge Technology) and focused onto the specimen via a low NA illumination objective (5 x 0.16NA, Zeiss EC Plan-Neofluar). The detection arm consisted of a water-immersion objective (20 x 1 NA, XLUMPlanFL, Olympus) mounted vertically onto a piezo nanopositioner (Piezosystem Jena MIPOS 500) allowing alignment of the focus plane with the light sheet. The fluorescence light was collected by a tube lens (150 mm focal length, Thorlabs AC254-150-A) and passed through a notch filter (NF488-15, Thorlabs) to eliminate 488 nm photons. The image was formed on a sCMOS sensor (PCO.edge 4.2, PCO). The 20x magnification yielded a field of view of 0.8 x 0.8 mm^2^ with a pixel dimension of 0.39μm^2^. The detection arm and specimen chamber were mounted on two independent XY translation stages to allow precise alignment of the specimen, detection axis and light sheet.

#### Imaging

Nonanesthetized *Tg(elavl3b*:*GCaMP6F*) larvae, 5 days post fertilisation, were immobilized at in 2.5% low melting point agarose (Sigma-Aldrich) prepared in Danieau solution and mounted dorsal side up on a raised glass platform that was placed in a custom-made Danieau-filled chamber. Pentylenetetrazole (Sigma-Aldrich) was added to the Danieau-filled chamber after 30 minutes of baseline imaging to a final concentration of 20 mM. Functional time-series were acquired at a rate of 20 Hz, 4x4 pixel binning (1.6 μm x 1.6 μm resolution). In order to achieve the maximum temporal resolution in these recordings, we have restricted imaging to the single plane, allowing a sampling frequency of 20Hz. The light sheet displays a hyperbolic profile along the light propagation axis. The diffraction-limited minimum (z-dimension) thickness of the light sheet (characterized by imaging 100 nm diameter fluorescent beads) was ~2.5 μm at the focal plane of the illumination objective. This value to increases to ~9 μm at a distance of 80 μm from the waist. This yields single neuron resolution over a field of view of ≃160 × 1000 μm centred on the midline of the larval fish- a region which contains the majority of neuronal cell bodies. At the extreme lateral margins of the fish the illumination sheet spans >1 neuron cell body dimeter and therefore does not provide single neuron resolution in these regions.

Time-series were aligned to a mean image of the functional imaging data for each fish (rigid body transformation as implemented in: SPM12, http://www.fil.ion.ucl.ac.uk/spm/software/spm12). Mean fluorescence traces were then extracted from ten anatomically defined regions of interest for further analyses. Analogous to other calcium-based connectivity in model organisms [62] anatomical regions were selected according to well-defined landmarks corresponding to the following regions in a publicly available standard zebrafish atlas (Z Brain Atlas, https://engertlab.fas.harvard.edu/Z-Brain [63], Cross-sectional images from the atlas corresponding to the structures included below are provided in **S1 Fig**):

- Tectum: Tectum Stratum Periventriculare, Tectum Neuropil
- Cerebellum: Cerebellum
- Rostral Hindbrain: Rhombomere 2, Rhombomere 3
- Mid Hindbrain: Rhombomere 4, Rhombomere 5, Rhombomere 6
- Caudal Hindbrain / Rostral Spinal Cord: Rhombomere 7, Spinal Cord

### Quantification and statistical analysis

#### Estimation of spectral data features

Mean fluorescence traces from the regions of interest were treated as multichannel time series for subsequent analysis. Short segments derived from a sliding window (length: 60s, step size: 10s) were used to estimate time-varying changes in the spectral composition of the time series: For each step of the sliding window the real component of the Fourier spectrum was calculated. A correlation matrix of region-specific mean Fourier amplitude across all time point was used to visualise slow fluctuations in distributed activity [64,65]. For the correlation, we calculated a vector containing the average power over the 0-10Hz frequency band of all channels, separately for each time window. We then calculate the full correlation matrix of each power-distribution vectors with the vectors at each other time point, yielding a *k*×*k* correlation matrix, where *k* is the total number of time steps.

Averages of the windowed Fourier spectra and the power correlation matrix across the studied animals are shown.

#### Simulated calcium imaging traces

To test the construct validity of the inversion approach, we used a neural mass model with known parameterisation to generate an LFP output, convolved this output with a calcium-imaging kernel, and inverted a DCM on those synthetic calcium-imaging traces to test whether the original parameterisation can be reconstructed.

The model was a standard prior three-source neural mass model implemented as ‘LFP’ model in the SPM12 model library [43]. We generated 6 segments of LFP-like model output with linear variation of a single parameter (*H*_*1*_) from *-1* to *+1*. The convolution kernel was constructed from a fast, inverted quadratic rise lasting *t*_*up*_ = 250*ms* of the form: *y*(*t*) = 2*t* * *t*_*up*_ − *t*^2^. This is followed by an exponential decay function of the form: $y(t)=e^{-\frac{1}{1000}t}$. Both functions were normalised so that *y*(*t*_*up*_) = 1. Parameters of these functions were chosen to approximate the reported dynamics of GCaMP6F [36].

#### Inversion of simulated calcium imaging traces

Each individual synthetic calcium imaging trace was inverted using a DCM for cross spectral densities approach with a single three-population neural mass model [66]. The DCM analysis relies on spectral features, which are estimated using a multivariate autoregressive model that provides (complex) cross-spectral densities for each time window and each fish separately. Each of these full cross spectra is then approximated during the Bayesian model inversion. We show an example of the spectral data features (here for a single spectral eigenmode in a single fish), and the corresponding model fits (**Fig 4A**)

Using a parametric empirical Bayes approach, we then compared the evidence for models, where changes in a single one of the parameter explain the difference between segments [67,68]. Parameter estimates for the winning parameter are then compared to the ‘ground truth’ parameter changes originally introduced into the generative model, therefore providing evidence for which parameter is changed, and how that parameter is changed to achieve the spectral changes contained in the time series.

#### Dynamic causal modelling of empirical calcium imaging traces

Baseline architecture: To characterise functional network architecture at rest, in an initial step only baseline data were analysed using a DCM approach. Specifically, 4-minute segments prior to PTZ were inverted using a single fully connected DCM containing 10 standard prior ‘LFP’ type sources [43]. DCM estimated parameter values for each of the directed, extrinsic (between region) coupling parameters, each of the intrinsic (within region) coupling parameters, regionally-specific time constants, as well as free energy approximations for the model evidence for the full model in each individual fish. Based on the full model inversion, smaller subsets of models were then compared with computational efficiency using Bayesian model reduction, which allows Bayesian model selection for the network architecture that best explains the baseline data [68]. The model space was designed as a full factorial design around three main features: the presence or absence of hierarchical connections between neighbouring brain regions (2 model families); the presence or absence of homologous connections between bilateral brain regions (2 model families); and the presence or absence of hub-like connections from one set of brain regions to all other regions (6 model families). Thus, the model evidence was estimated across 2*2*6 = 24 model types, and evaluated using family-wise Bayesian model comparison across sections of this full model space to yield inference about hub-type connections (comparing 6 model families), and short-range connections (comparing 2 * 2 = 4 model families). The null model (Model 0) is a model where no between-region (i.e., extrinsic connectivity) exists and is included for completeness. In this model, each node is equipped with its own steady state input (a parameterised pink noise function), simulating background local activity, and has several free intrinsic connectivity parameters (local connection strengths H_1-5_ and time constants of local connectivity T_E_ and T_I_) (Fig 3). This model space was chosen to emulate some basic features of brain connectivity found across many species and systems, specifically rich-club organisation (modelled as hub-like connectivity), hierarchical message passing (modelled as forward/backward connections along neighbouring nodes), and typically seen homotopic connections between symmetrical structures.

Seizure data inversion: Based on the dynamic network architecture identified in the step above, an additional DCM analysis was performed to identify slow fluctuations of synaptic parameters within this architecture that could explain seizure activity. For this, data were again divided into segments using a sliding window approach (60s, 50s steps) for each animal separately. DCMs with the architecture derived from the step above were inverted separately for each individual time window.

We then constructed a second level model to estimate between-time window variations in parameters using a parametric empirical Bayesian approach [29,68]. This contained several temporal basis functions that in combination can explain a majority of possible parameter trajectories: (1) an ‘on/off’ tonic seizure effect step function with onset at PTZ injection; (2) a monophasic seizure effect function with onset at PTZ injection; (3) a linear increase with onset at PTZ injection; (4) a set of three discrete cosine basis functions to model; (5) a set of three regressors modelling random between-fish effects.

This approach provides estimates for how between-time window parameter changes can be modelled as a linear combination of the basis sets provided, as well as a free energy estimate for the model evidence. We can thus perform Bayesian model reduction and selection at this second level, comparing competing model families where only subsets of parameters are free to vary between time windows, and thus select a subset of parameters that best explain the observed changes over time. We broadly divided the model space of these between time-window (i.e. between individual DCM) effects into (a) models with variations in hierarchical coupling, (b) models with variations in hub coupling, and (c) models with variations in intrinsic synaptic coupling parameters as outlined in Fig 4. Family-wise Bayesian model selection was used to select relevant parameters, which were freed in a single model to provide parameter estimates at time window with the estimated maximum PTZ effect.

Forward modelling: To further explore the effects of specific parameter changes, the optic tectum with its hub-like position in the network was analysed further. Posterior parameter estimates for each time windows derived from the PEB-DCM analysis above were grouped into time constant and connection strength changes. In order to allow a low dimensional projection of the multiple parameters of interest we performed a principal component analysis separately on the intrinsic connectivity parameters (H_1-5_); and the time constant parameters (T_E_, T_I_). The first principal component of each of these categories was then used to project the parameter changes into a two-dimensional plane. Because the DCM provides a fully generative model, we can not only plot the parameter estimates, but also generate a predicted spectral output for each point across this plane, by adding the respective principal component values to the baseline parameterisation of the model and simulating its output. We plotted the resultant low frequency (delta-range), and high frequency (gamma-range) power across the parameter space, to indicate how movement in parameter space affects the spectral output.

### Data and software availability

#### Software

Analysis in this study was built on tools available as part of the academic freeware package ‘Statistical Parametric Mapping 12’ (www.fil.ion.ucl.ac.uk/spm). This toolbox and all custom code runs on Mathworks Matlab (https://uk.mathworks.com/products/matlab.html). Custom code is freely available as a github repository (http://github.com/roschkoenig/Zebrafish_Seizure).

#### Data resources

All data used in this analysis are available online in an Open Science Framework repository (http://doi.org/10.17605/OSF.IO/Q7KTH). This contains full length recordings (as time series), as well as windowed data used for DCM analysis.

## Supporting information

S1 Fig Atlas regions corresponding to the anatomical segmentation used in our analysis (all images at z = -90. (A) ‘Tectum’ corresponds to Z Brain regions Tectum Stratum Periventriculare and Tectum Neuropil. (B) ‘Cerebellum’ corresponds to Z Brain region Cerebellum. (C) ‘Rostral Hindbrain’ corresponds to Z Brain regions Rhombomere 2 and Rhombomere 3. (D) ‘Mid Hindbrain’ corresponds to Z Brain region Rhombomere 4, Rhombomere 5, and Rhombomere 6. (E) ‘Caudal Hindbrain / Rostral Spinal Cord’ corresponds to Z Brain regions Rhombomere 7, and Spinal Cord. Images are taken from https://engertlab.fas.harvard.edu/Z-Brain [accessed 18/05/2018].
(DOCX)

S2 Fig (A) Fourier spectra are shown for light sheet recordings of individual animal recordings sessions. Using a sliding window (size 60s, step 10s), windowed estimates are made of the frequency composition of the mean time fluorescence time series across all regions and plotted over time with colour-coding indicating the power at particular frequencies. Seizure onset is associated with increase in low frequency activty. The early ictal period is characterised by frequent broadband frequency bursts, which become less frequent in the late ictal state. (B) Frequency power plots are shown for individual regions at preictal, early ictal and late ictal intervals for each fish. Colours indicate the brain region as indicated by the key. Individual fish show reproducible patterns of localised frequency power distribution changes at seizure onset–in the preictal state the caudal hindbrain / rostral spinal cord (CHbr/RSc) show highest overall activity; during early and late seizure activity in the rostral hindbrain (RHbr) has the highest broadband power.
(DOCX)

S3 Fig Graphs show the full cross-spectral density spectra predicted from the dynamic causal models derived from the hierarchical model inversion across all time windows and fish. Each graph shows time-windowed power spectral density estimates for the optic tectum, with colours indicating the time of the experiment. Each fish shows recognisable frequency peaks at approximately 20Hz, and 90Hz, which differ quantitatively. Note that the high frequency peak is predicted to achieve its maximum just after PTZ injection for each of the fish.
(DOCX)

S4 Fig Analogous to Fig 5 in the main text, this figure shows a low dimensional projection of the parameter values for each individual time window as estimated for the optic tectum. Here we plot an additional third dimension (the second component of the PCA over the connectivity strengths), revealing a clearer separation of the different seizure phases, indicating the transition from pre-ictal, to early seizure, to late seizure phases. [The colormap corresponds to main Fig 5].
(DOCX)

## References

[1] Thomas RH, Berkovic SF. The hidden genetics of epilepsy—a clinically important new paradigm. Nat Rev Neurol. 2014; 10(5):283–92. 10.1038/nrneurol.2014.62 24733163

[2] Parker L, Padilla M, Du Y, Dong K, Tanouye M a. Drosophila as a model for epilepsy: bss is a gain-of-function mutation in the para sodium channel gene that leads to seizures. Genetics. 2011; 187(2):523–34. 10.1534/genetics.110.123299 21115970 PMC3030494

[3] Depaulis A, David O, Charpier S. The genetic absence epilepsy rat from Strasbourg as a model to decipher the neuronal and network mechanisms of generalized idiopathic epilepsies. J Neurosci Methods. 2015;:1–16. 10.1016/j.jneumeth.2015.05.022 26068173

[4] Seo S, Leitch B. Altered thalamic GABAA-receptor subunit expression in the stargazer mouse model of absence epilepsy. Epilepsia. 2014; 55(2):224–32. 10.1111/epi.12500 24417662

[5] Dhindsa RS, Goldstein DB. Genetic Discoveries Drive Molecular Analyses and Targeted Therapeutic Options in the Epilepsies. Curr Neurol Neurosci Rep. 2015; 15(10):70 10.1007/s11910-015-0587-4 26319171

[6] Baraban SC, Dinday MT, Hortopan GA. Drug screening in Scn1a zebrafish mutant identifies clemizole as a potential Dravet syndrome treatment. Nat Commun. 2013; 4:2410 10.1038/ncomms3410 24002024 PMC3891590

[7] Griffin A, Hamling KR, Knupp K, Hong S, Lee LP, Baraban SC. Clemizole and modulators of serotonin signalling suppress seizures in Dravet syndrome. Brain. 2017;:aww342. 10.1093/brain/aww342 PMC6075536 28073790

[8] Kibat C, Krishnan S, Ramaswamy M, Baker BJ, Jesuthasan S. Imaging voltage in zebrafish as a route to characterizing a vertebrate functional connectome: promises and pitfalls of genetically encoded indicators. J Neurogenet. 2016; 30(2):80–8. 10.1080/01677063.2016.1180384 27328843

[9] Ahrens MB, Orger MB, Robson DN, Li JM, Keller PJ. Whole-brain functional imaging at cellular resolution using light-sheet microscopy. Nat Methods. 2013; 10(5):413–20. 10.1038/nmeth.2434 23524393

[10] Afrikanova T, Serruys ASK, Buenafe OEM, Clinckers R, Smolders I, de Witte P a M, et al Validation of the Zebrafish Pentylenetetrazol Seizure Model: Locomotor versus Electrographic Responses to Antiepileptic Drugs. PLoS One. 2013; 8(1):1–9. 10.1371/journal.pone.0054166 PMC3544809 23342097

[11] Winter MJ, Redfern WS, Hayfield AJ, Owen SF, Valentin J-P, Hutchinson TH. Validation of a larval zebrafish locomotor assay for assessing the seizure liability of early-stage development drugs. J Pharmacol Toxicol Methods. 2008; 57(3):176–87. 10.1016/j.vascn.2008.01.004 18337127

[12] Meyer M, Dhamne SC, LaCoursiere CM, Tambunan D, Poduri A, Rotenberg A. Microarray noninvasive neuronal seizure recordings from intact larval zebrafish. PLoS One. 2016; 11(6):1–17. 10.1371/journal.pone.0156498 PMC4900632 27281339

[13] Hong S, Lee P, Baraban SC, Lee LP. A Novel Long-term, Multi-Channel and Non-invasive Electrophysiology Platform for Zebrafish. Sci Rep. 2016; 6(January):28248. 27305978 10.1038/srep28248 PMC4910293

[14] Turrini L, Fornetto C, Marchetto G, M?llenbroich MC, Tiso N, Vettori A, et al Optical mapping of neuronal activity during seizures in zebrafish. Sci Rep. 2017; 7(1):3025 10.1038/s41598-017-03087-z 28596596 PMC5465210

[15] Winter MJ, Windell D, Metz J, Matthews P, Pinion J, Brown JT, et al 4-Dimensional Functional Profiling in the Convulsant-Treated Larval Zebrafish Brain. Sci Rep. 2017; 7(1):6581 10.1038/s41598-017-06646-6 28747660 PMC5529444

[16] Lopes da Silva FH, Hoeks A, Smits H, Zetterberg LH. Model of brain rhythmic activity. The alpha-rhythm of the thalamus. Kybernetik. 1974; 15(1):27–37. 4853232 10.1007/BF00270757

[17] Jansen BH, Rit VG. Electroencephalogram and visual evoked potential generation in a mathematical model of coupled cortical columns. Biol Cybern. 1995; 73(4):357–66. 7578475 10.1007/BF00199471

[18] Lytton WW. Computer modelling of epilepsy. Nat Rev Neurosci. 2008; 9(8):626–37. 10.1038/nrn2416 18594562 PMC2739976

[19] Jirsa VK, Stacey WC, Quilichini PP, Ivanov AI, Bernard C. On the nature of seizure dynamics. Brain. 2014; 137(8):2210–30. 24919973 10.1093/brain/awu133 PMC4107736

[20] Friston KJ, Harrison L, Penny W. Dynamic causal modelling. Neuroimage. 2003; 19(4):1273–302. 12948688 10.1016/s1053-8119(03)00202-7

[21] Wang Y, Goodfellow M, Taylor PN, Baier G. Phase space approach for modeling of epileptic dynamics. Phys Rev E. 2012; 85(6):61918. 10.1103/PhysRevE.85.061918 23005138

[22] Goodfellow M, Schindler K, Baier G. Self-organised transients in a neural mass model of epileptogenic tissue dynamics. Neuroimage. 2012; 59(3):2644–60. 10.1016/j.neuroimage.2011.08.060 21945465

[23] Kameneva T, Ying T, Guo B, Freestone DR. Neural mass models as a tool to investigate neural dynamics during seizures. J Comput Neurosci. 2017; 10.1007/s10827-017-0636-x 28102460

[24] Blenkinsop A, Valentin A, Richardson MP, Terry JR. The dynamic evolution of focal-onset epilepsies—combining theoretical and clinical observations. Eur J Neurosci. 2012; 36(2):2188–200. 10.1111/j.1460-9568.2012.08082.x 22805064

[25] Schiff SJ, Sauer T. Kalman filter control of a model of spatiotemporal cortical dynamics. J Neural Eng. 2008; 5:1–8. 10.1088/1741-2560/5/1/001 18310806 PMC2276637

[26] Lie O V., van Mierlo P. Seizure-Onset Mapping Based on Time-Variant Multivariate Functional Connectivity Analysis of High-Dimensional Intracranial EEG: A Kalman Filter Approach. Brain Topogr. 2017; 30(1):46–59. 10.1007/s10548-016-0527-x 27722839

[27] Cooray GK, Sengupta B, Douglas PK, Friston K. Dynamic causal modelling of electrographic seizure activity using Bayesian belief updating. Neuroimage. 2016; 125:1142–54. 10.1016/j.neuroimage.2015.07.063 26220742 PMC4692455

[28] Papadopoulou M, Leite M, van Mierlo P, Vonck K, Lemieux L, Friston K, et al Tracking slow modulations in synaptic gain using dynamic causal modelling: Validation in epilepsy. Neuroimage. 2015; 107:117–26. 10.1016/j.neuroimage.2014.12.007 25498428 PMC4306529

[29] Papadopoulou M, Cooray G, Rosch R, Moran R, Marinazzo D, Friston K. Dynamic causal modelling of seizure activity in a rat model. Neuroimage. 2016; 10.1016/j.neuroimage.2016.08.062 27639356

[30] Breakspear M. A Unifying Explanation of Primary Generalized Seizures Through Nonlinear Brain Modeling and Bifurcation Analysis. Cereb Cortex. 2005; 16(9):1296–313. 10.1093/cercor/bhj072 16280462

[31] Omidvarnia A, Pedersen M, Rosch RE, Friston KJ, Jackson GD. Hierarchical disruption in the Bayesian brain: Focal epilepsy and brain networks. NeuroImage Clin. 2017; 10.1016/j.nicl.2017.05.019 PMC5486238 28702345

[32] Sinha N, Dauwels J, Kaiser M, Cash SS, Westover MB, Wang Y, et al. Predicting neurosurgical outcomes in focal epilepsy patients using computational modelling. 2016; 10.1093/brain/aww299 PMC5278304 28011454

[33] Nehlig A. Mapping of neuronal networks underlying generalized seizures induced by increasing doses of pentylenetetrazol in the immature and adult rat: A c-Fos immunohistochemical study. Eur J Neurosci. 1998; 10(6):2094–106. 9753096 10.1046/j.1460-9568.1998.00223.x

[34] Burns SP, Santaniello S, Yaffe RB, Jouny CC, Crone NE, Bergey GK, et al Network dynamics of the brain and influence of the epileptic seizure onset zone. Proc Natl Acad Sci. 2014; 111(49):E5321–30. 10.1073/pnas.1401752111 25404339 PMC4267355

[35] Netoff TI, Clewley R, Arno S, Keck T, White J a. Epilepsy in Small-World Networks. J Neurosci. 2004; 24(37):8075–83. 10.1523/JNEUROSCI.1509-04.2004 15371508 PMC6729784

[36] Chen T-W, Wardill TJ, Sun Y, Pulver SR, Renninger SL, Baohan A, et al Ultrasensitive fluorescent proteins for imaging neuronal activity. Nature. 2013; 499(7458):295–300. 10.1038/nature12354 23868258 PMC3777791

[37] Cooray GK, Sengupta B, Douglas P, Englund M, Wickstrom R, Friston K. Characterising seizures in anti-NMDA-receptor encephalitis with dynamic causal modelling. Neuroimage. 2015; 118:508–19. 10.1016/j.neuroimage.2015.05.064 26032883 PMC4558461

[38] Huang RQ, Bell-Horner CL, Dibas MI, Covey DF, Drewe J a, Dillon GH. Pentylenetetrazole-induced inhibition of recombinant gamma-aminobutyric acid type A (GABA(A)) receptors: mechanism and site of action. J Pharmacol Exp Ther. 2001; 298(3):986–95. 11504794

[39] Kalueff AV. Mapping convulsants’ binding to the GABA-A receptor chloride ionophore: A proposed model for channel binding sites. Neurochem Int. 2007; 50(1):61–8. 10.1016/j.neuint.2006.07.004 16959376 PMC1939818

[40] Baraban SC, Taylor MR, Castro PA, Baier H. Pentylenetetrazole induced changes in zebrafish behavior, neural activity and c-fos expression. NSC. 2005; 131(3):759–68. 10.1016/j.neuroscience.2004.11.031 15730879

[41] Chan AW, Mohajerani MH, LeDue JM, Wang YT, Murphy TH. Mesoscale infraslow spontaneous membrane potential fluctuations recapitulate high-frequency activity cortical motifs. Nat Commun. 2015; 6:7738 10.1038/ncomms8738 26190168 PMC5101061

[42] Keller PJ, Ahrens MB, Freeman J. Light-sheet imaging for systems neuroscience. Nat Methods. 2014; 12(1):27–9. 10.1038/nmeth.3214 25549267

[43] Moran R, Pinotsis DA, Friston K. Neural masses and fields in dynamic causal modeling. Front Comput Neurosci. 2013; 7(May):57. 23755005 10.3389/fncom.2013.00057 PMC3664834

[44] Kuhlmann L, Grayden DB, Wendling F, Schiff SJ. Role of Multiple-Scale Modeling of Epilepsy in Seizure Forecasting. J Clin Neurophysiol. 2015; 32(3):220–6. 10.1097/WNP.0000000000000149 26035674 PMC4455036

[45] Rahmati V, Kirmse K, Markovi? D, Holthoff K, Kiebel SJ. Inferring Neuronal Dynamics from Calcium Imaging Data Using Biophysical Models and Bayesian Inference. Hilgetag CC, editor. PLOS Comput Biol. 2016; 12(2):e1004736 10.1371/journal.pcbi.1004736 26894748 PMC4760968

[46] Chen X, Mu Y, Hu Y, Kuan AT, Randlett O, Nikitchenko M, et al Brain-wide organization of neuronal activity in larval zebrafish. Submitt to Nat Methods. 2017;:1–38. 10.1016/j.neuron.2018.09.042 PMC6543271 30473013

[47] Lovett-Barron M, Andalman AS, Allen WE, Vesuna S, Kauvar I, Burns VM, et al Ancestral Circuits for the Coordinated Modulation of Brain State. Cell. 2017;:1–13. 10.1016/j.cell.2017.10.021 PMC5725395 29103613

[48] Niell CM, Smith SJ. Functional imaging reveals rapid development of visual response properties in the zebrafish tectum. Neuron. 2005; 45(6):941–51. 10.1016/j.neuron.2005.01.047 15797554

[49] Niell CM, Meyer MP, Smith SJ. In vivo imaging of synapse formation on a growing dendritic arbor. Nat Neurosci. 2004; 7(3):254–60. 10.1038/nn1191 14758365

[50] Meyer MP. Evidence from In Vivo Imaging That Synaptogenesis Guides the Growth and Branching of Axonal Arbors by Two Distinct Mechanisms. J Neurosci. 2006; 26(13):3604–14. 10.1523/JNEUROSCI.0223-06.2006 16571769 PMC6673851

[51] Portugues R, Engert F. The neural basis of visual behaviors in the larval zebrafish. Curr Opin Neurobiol. 2009; 19(6):644–7. 10.1016/j.conb.2009.10.007 19896836 PMC4524571

[52] Schindler K a., Bialonski S, Horstmann MT, Elger CE, Lehnertz K. Evolving functional network properties and synchronizability during human epileptic seizures. Chaos. 2008; 18(3). 10.1063/1.2966112 19045457

[53] Nam RH, Kim W, Lee CJ. NMDA receptor-dependent long-term potentiation in the telencephalon of the zebrafish. Neurosci Lett. 2004; 370(2–3):248–51. 10.1016/j.neulet.2004.08.037 15488332

[54] Proix T, Bartolomei F, Chauvel P, Bernard C, Jirsa VK. Permittivity Coupling across Brain Regions Determines Seizure Recruitment in Partial Epilepsy. J Neurosci. 2014; 34(45):15009–21. 10.1523/JNEUROSCI.1570-14.2014 25378166 PMC6608363

[55] Lee V, Maguire J. The impact of tonic GABAA receptor-mediated inhibition on neuronal excitability varies across brain region and cell type. Front Neural Circuits. 2014; 8(February):3. 24550784 10.3389/fncir.2014.00003 PMC3909947

[56] Chaudhuri R, Bernacchia A, Wang XJ. A diversity of localized timescales in network activity. Elife. 2014; 3:e01239 10.7554/eLife.01239 24448407 PMC3895880

[57] Koch C, Rapp M, Segev I. A brief history of time (constants). Cerebr Cortex. 1996; 6:93–101. 10.1093/cercor/6.2.93 8670642

[58] Rubinov M, Sporns O, Thivierge JP, Breakspear M. Neurobiologically realistic determinants of Self-Organized criticality in networks of spiking neurons. PLoS Comput Biol. 2011; 7(6). 10.1371/journal.pcbi.1002038 PMC3107249 21673863

[59] Shu Y, Hasenstaub A, McCormick D a. Turning on and off recurrent balanced cortical activity. Nature. 2003; 423(May):288–93. 12748642 10.1038/nature01616

[60] Dunn TW, Mu Y, Narayan S, Randlett O, Naumann E a., Yang CT, et al Brain-wide mapping of neural activity controlling zebrafish exploratory locomotion. Elife. 2016; 5(MARCH2016):1–29. 10.7554/eLife.12741 PMC4841782 27003593

[61] Wolf S, Supatto W, Debrégeas G, Mahou P, Kruglik SG, Sintes J-M, et al Whole-brain functional imaging with two-photon light-sheet microscopy. Nat Methods. 2015; 12(5):379–80. 10.1038/nmeth.3371 25924070

[62] Mann K, Gallen CL, Clandinin TR. Whole-Brain Calcium Imaging Reveals an Intrinsic Functional Network in Drosophila. Curr Biol. 2017; 27(15):2389–2396.e4. 10.1016/j.cub.2017.06.076 28756955 PMC5967399

[63] Randlett O, Wee CL, Naumann E a, Nnaemeka O, Schoppik D, Fitzgerald JE, et al Whole-brain activity mapping onto a zebrafish brain atlas. Nat Methods. 2015; 12(11):1039–46. 10.1038/nmeth.3581 26778924 PMC4710481

[64] Rosch RE, Baldeweg T, Moeller F, Baier G. Network Dynamics In The Healthy And Epileptic Developing Brain. bioRxiv. 2017; 10.1162/NETN_a_00026 PMC5989999 29911676

[65] Betzel RF, Erickson MA, Abell M, O’Donnell BF, Hetrick WP, Sporns O. Synchronization dynamics and evidence for a repertoire of network states in resting EEG. Front Comput Neurosci. 2012; 6(September):1–13. 23060785 10.3389/fncom.2012.00074 PMC3460532

[66] Moran RJ, Stephan KE, Dolan RJ, Friston KJ. Consistent spectral predictors for dynamic causal models of steady-state responses. Neuroimage. 2011; 55(4):1694–708. 10.1016/j.neuroimage.2011.01.012 21238593 PMC3093618

[67] Rosch RE, Cooray G, Friston KJ. Dynamic Causal Modelling of Dynamic Dysfunction in NMDA-Receptor Antibody Encephalitis. In: 2017; Springer, Cham; p. 121–48.

[68] Friston KJ, Litvak V, Oswal A, Razi A, Stephan KE, van Wijk BCM, et al Bayesian model reduction and empirical Bayes for group (DCM) studies. Neuroimage. 2016; 128:413–31. 10.1016/j.neuroimage.2015.11.015 26569570 PMC4767224

