# Supplementary material for: Calcium imaging and dynamic causal modelling reveal brain-wide changes in effective connectivity and synaptic dynamics during epileptic seizures
Source: PLoS Comput Biol. 2018 Aug 23;14(8):e1006375. doi: 10.1371/journal.pcbi.1006375 (PMC6124808; doi:10.1371/journal.pcbi.1006375)
Supplement: S1 Fig — (A) ‘Tectum’ corresponds to Z Brain regions Tectum Stratum Periventriculare and Tectum Neuropil. (B) ‘Cerebellum’ corresponds to Z Brain region Cerebellum. (C) ‘Rostral Hindbrain’ corresponds to Z Brain regions Rhombomere 2 and Rhombomere 3. (D) ‘Mid Hindbrain’ corresponds to Z Brain region Rhombomere 4, Rhombomere 5, and Rhombomere 6. (E) ‘Caudal Hindbrain / Rostral Spinal Cord’ corresponds to Z Brain regions Rhombomere 7, and Spinal Cord. Images are taken from https://engertlab.fas.harvard.edu/Z-Brain [accessed 18/05/2018]. (DOCX) [file pcbi.1006375.s001.docx]

| **A**  **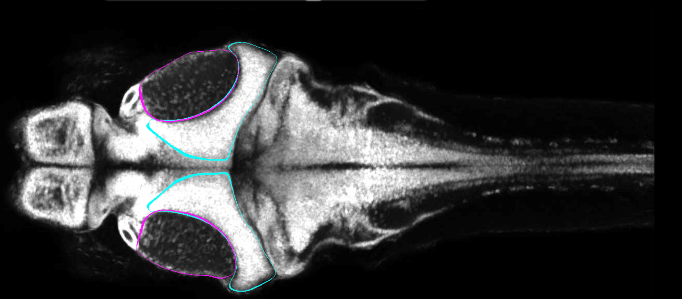** | **B**  **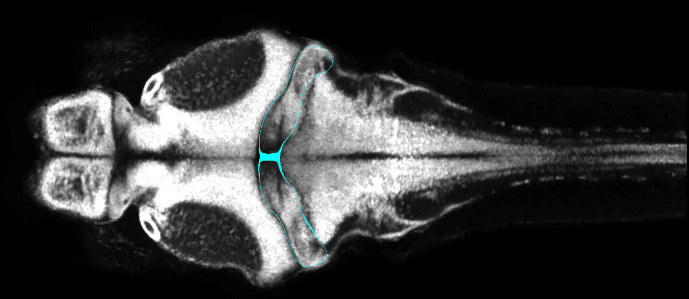** |
| --- | --- |
| **C**  **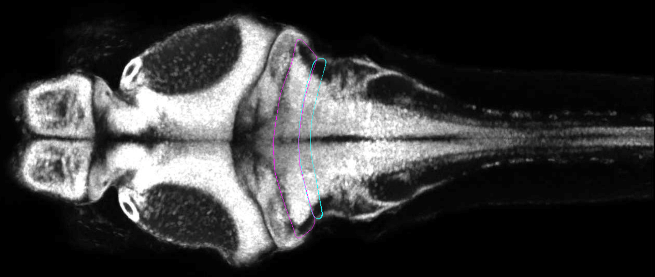** | **D**  **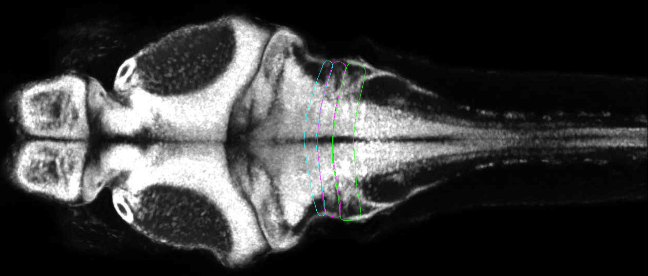** |
|  |  |
| **E**  **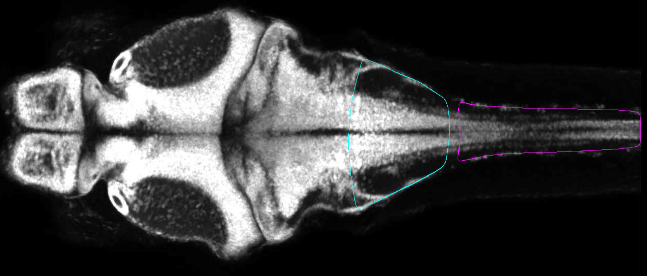** |  |
| **Supplementary Figure S1:** **Atlas regions corresponding to the anatomical segmentation used in our** analysis (all images at z = -90. (A) ‘Tectum’ corresponds to Z Brain regions Tectum Stratum Periventriculare and Tectum Neuropil. (B) ‘Cerebellum’ corresponds to Z Brain region Cerebellum. (C) ‘Rostral Hindbrain’ corresponds to Z Brain regions Rhombomere 2 and Rhombomere 3. (D) ‘Mid Hindbrain’ corresponds to Z Brain region Rhombomere 4, Rhombomere 5, and Rhombomere 6. (E) ‘Caudal Hindbrain / Rostral Spinal Cord’ corresponds to Z Brain regions Rhombomere 7, and Spinal Cord. Images are taken from <https://engertlab.fas.harvard.edu/Z-Brain> [accessed 18/05/2018]. | |
