# Supplementary material for: Calcium imaging and dynamic causal modelling reveal brain-wide changes in effective connectivity and synaptic dynamics during epileptic seizures
Source: PLoS Comput Biol. 2018 Aug 23;14(8):e1006375. doi: 10.1371/journal.pcbi.1006375 (PMC6124808; doi:10.1371/journal.pcbi.1006375)
Supplement: S2 Fig — (A) Fourier spectra are shown for light sheet recordings of individual animal recordings sessions. Using a sliding window (size 60s, step 10s), windowed estimates are made of the frequency composition of the mean time fluorescence time series across all regions and plotted over time with colour-coding indicating the power at particular frequencies. Seizure onset is associated with increase in low frequency activty. The early ictal period is characterised by frequent broadband frequency bursts, which become less frequent in the late ictal state. (B) Frequency power plots are shown for individual regions at preictal, early ictal and late ictal intervals for each fish. Colours indicate the brain region as indicated by the key. Individual fish show reproducible patterns of localised frequency power distribution changes at seizure onset–in the preictal state the caudal hindbrain / rostral spinal cord (CHbr/RSc) show highest overall activity; during early and late seizure activity in the rostral hindbrain (RHbr) has the highest broadband power. (DOCX) [file pcbi.1006375.s002.docx]

| 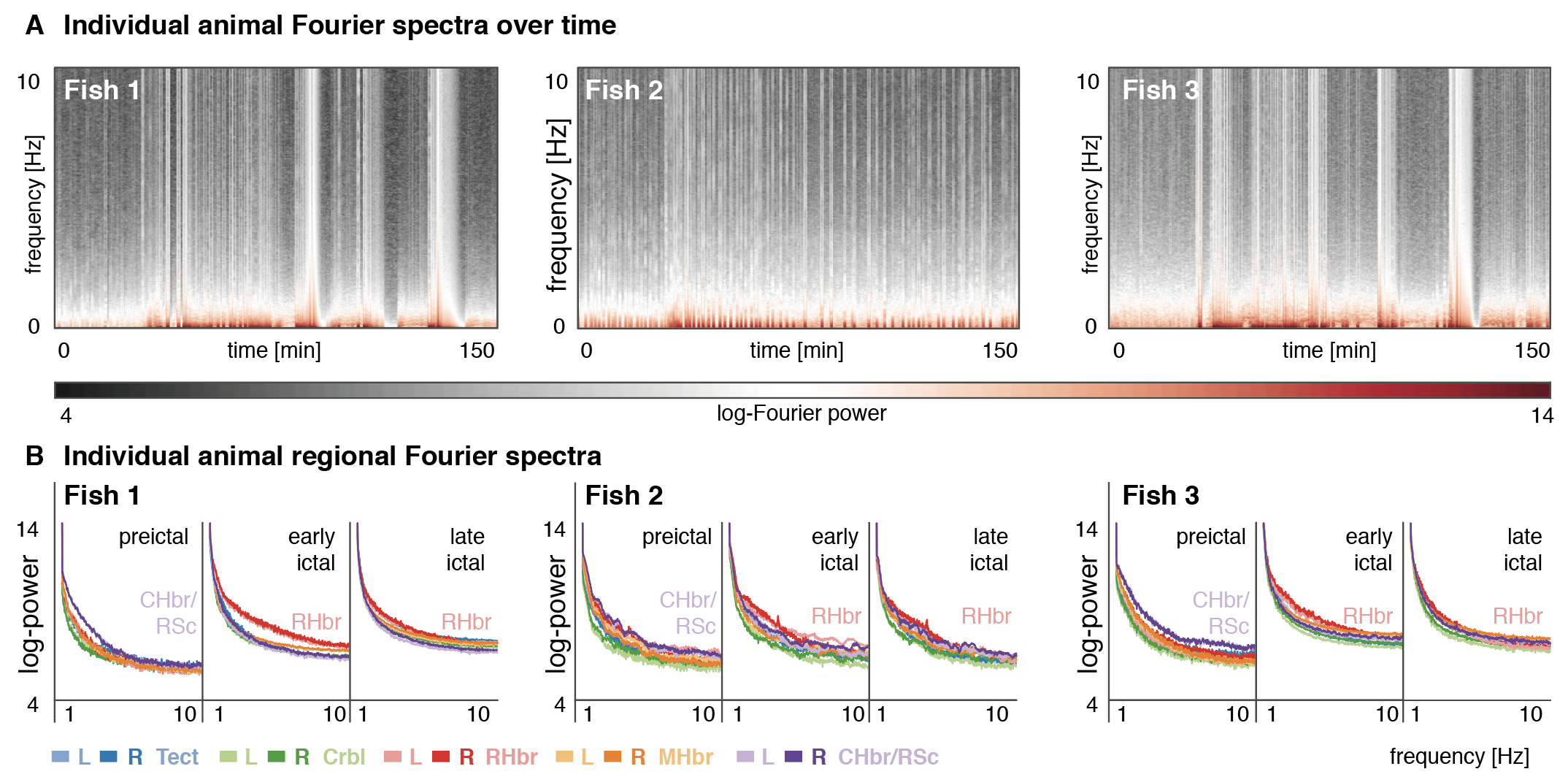 |
| --- |
| **Supplementary Figure S2:** (A) Fourier spectra are shown for light sheet recordings of individual animal recordings sessions. Using a sliding window (size 60s, step 10s), windowed estimates are made of the frequency composition of the mean time fluorescence time series across all regions and plotted over time with colour-coding indicating the power at particular frequencies. Seizure onset is associated with increase in low frequency activty. The early ictal period is characterised by frequent broadband frequency bursts, which become less frequent in the late ictal state. (B) Frequency power plots are shown for individual regions at preictal, early ictal and late ictal intervals for each fish. Colours indicate the brain region as indicated by the key. Individual fish show reproducible patterns of localised frequency power distribution changes at seizure onset – in the preictal state the caudal hindbrain / rostral spinal cord (CHbr/RSc) show highest overall activity; during early and late seizure activity in the rostral hindbrain (RHbr) has the highest broadband power. |
