# Supplementary material for: Calcium imaging and dynamic causal modelling reveal brain-wide changes in effective connectivity and synaptic dynamics during epileptic seizures
Source: PLoS Comput Biol. 2018 Aug 23;14(8):e1006375. doi: 10.1371/journal.pcbi.1006375 (PMC6124808; doi:10.1371/journal.pcbi.1006375)
Supplement: S3 Fig — Each graph shows time-windowed power spectral density estimates for the optic tectum, with colours indicating the time of the experiment. Each fish shows recognisable frequency peaks at approximately 20Hz, and 90Hz, which differ quantitatively. Note that the high frequency peak is predicted to achieve its maximum just after PTZ injection for each of the fish. (DOCX) [file pcbi.1006375.s003.docx]

| 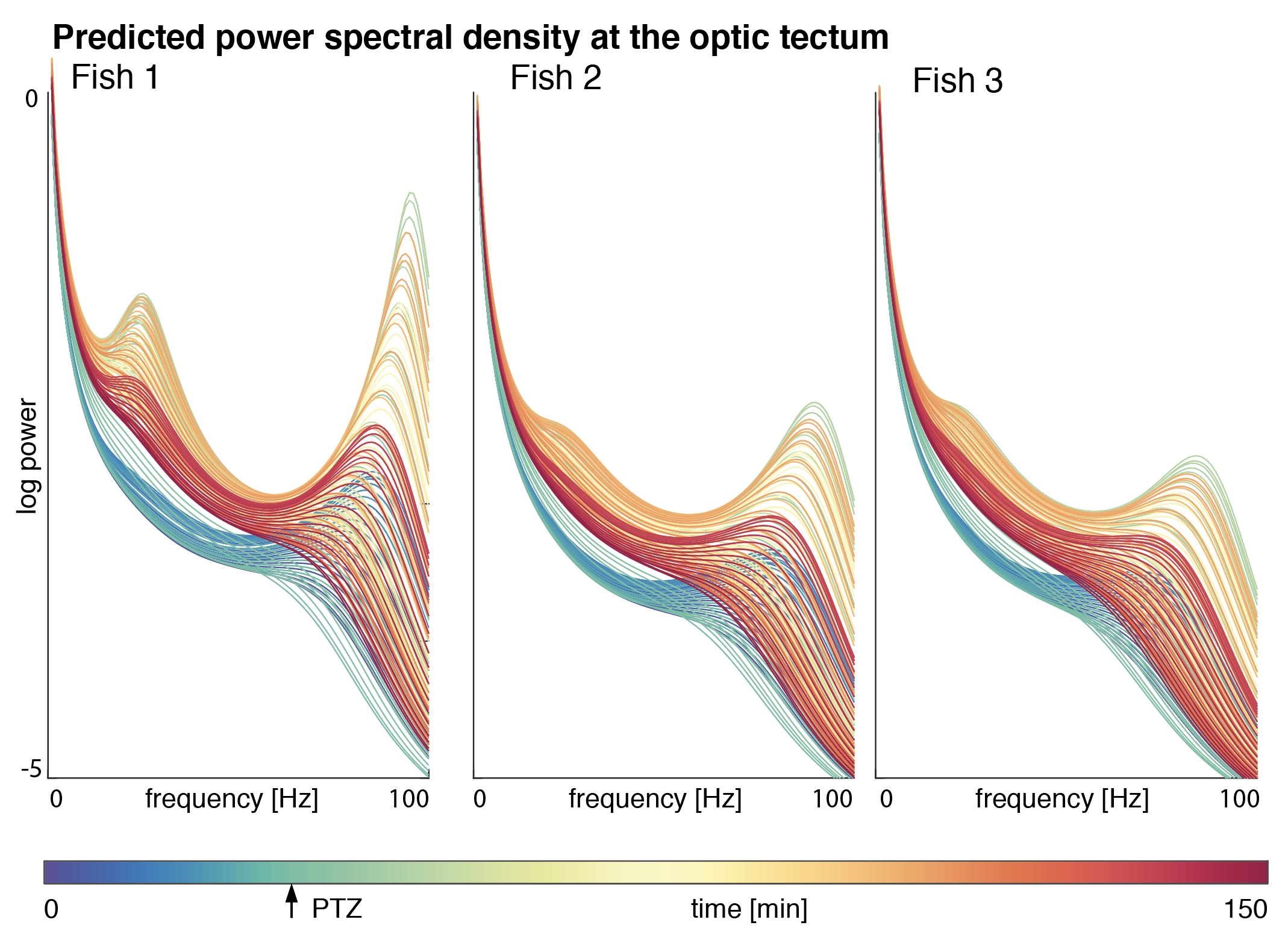 |
| --- |
| **Supplemental Figure S3:** Graphs show the full cross-spectral density spectra predicted from the dynamic causal models derived from the hierarchical model inversion across all time windows and fish. Each graph shows time-windowed power spectral density estimates for the optic tectum, with colours indicating the time of the experiment. Each fish shows recognisable frequency peaks at approximately 20Hz, and 90Hz, which differ quantitatively. Note that the high frequency peak is predicted to achieve its maximum just after PTZ injection for each of the fish. |
