# Supplementary material for: Calcium imaging and dynamic causal modelling reveal brain-wide changes in effective connectivity and synaptic dynamics during epileptic seizures
Source: PLoS Comput Biol. 2018 Aug 23;14(8):e1006375. doi: 10.1371/journal.pcbi.1006375 (PMC6124808; doi:10.1371/journal.pcbi.1006375)
Supplement: S4 Fig — Here we plot an additional third dimension (the second component of the PCA over the connectivity strengths), revealing a clearer separation of the different seizure phases, indicating the transition from pre-ictal, to early seizure, to late seizure phases. [The colormap corresponds to main Fig 5]. (DOCX) [file pcbi.1006375.s004.docx]

| 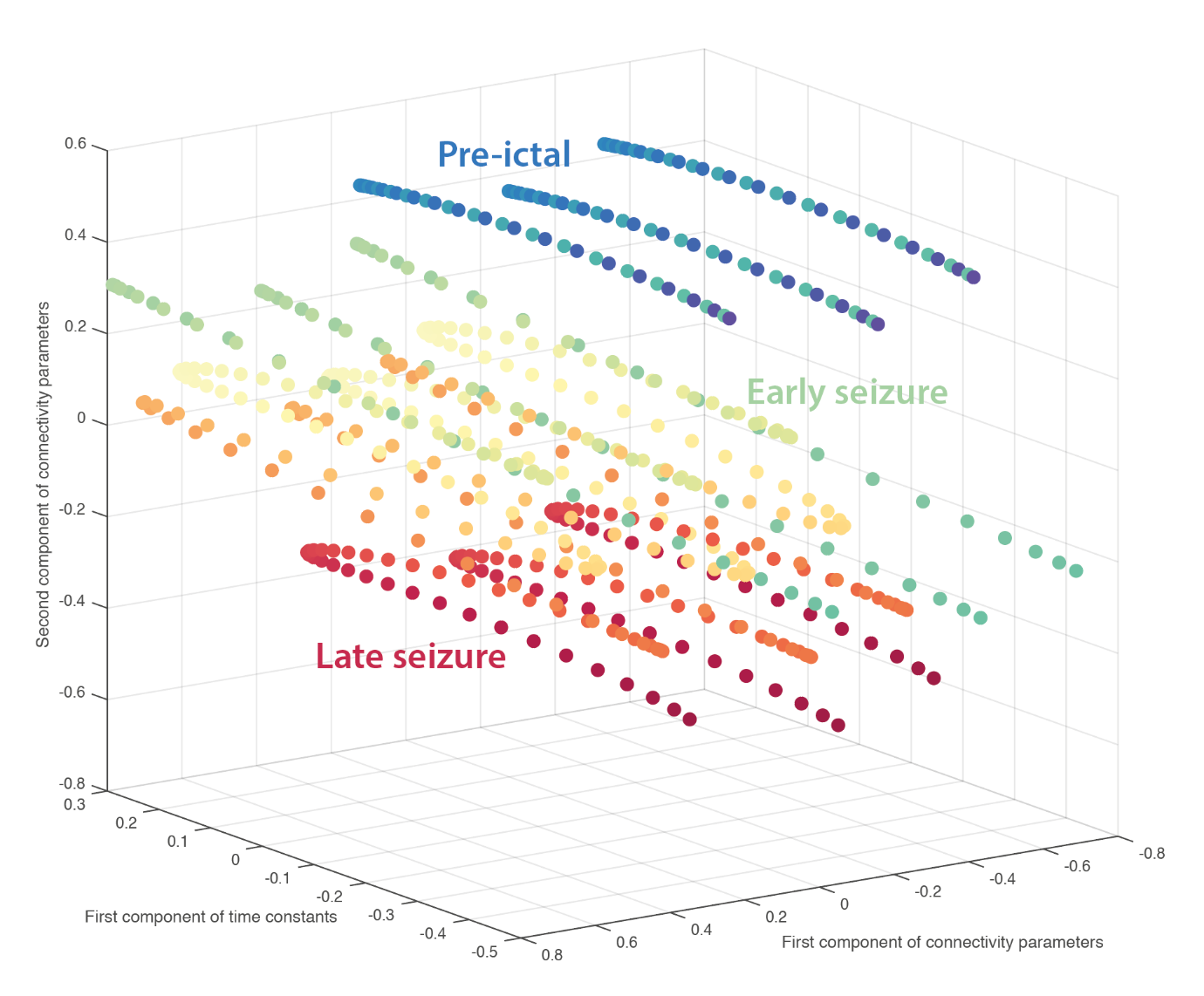 |
| --- |
| **Supplemental Figure 4:** Analogous to Figure 5 in the main text, this figure shows a low dimensional projection of the parameter values for each individual time window as estimated for the optic tectum. Here we plot an additional third dimension (the second component of the PCA over the connectivity strengths), revealing a clearer separation of the different seizure phases, indicating the transition from pre-ictal, to early seizure, to late seizure phases. [The colormap corresponds to main Figure 5] |
